# Understanding the role of the gut in undernutrition: what can technology tell us?

**DOI:** 10.1136/gutjnl-2020-323609

**Published:** 2021-06-08

**Authors:** Alex J Thompson, Claire D Bourke, Ruairi C Robertson, Nirupama Shivakumar, Christine A Edwards, Tom Preston, Elaine Holmes, Paul Kelly, Gary Frost, Douglas J Morrison, HUNGer Consortium

**Affiliations:** 1 Hamlyn Centre for Robotic Surgery, Department of Surgery and Cancer, Imperial College London, London, UK; 2 Blizard Institute, Barts & The London School of Medicine, Queen Mary University of London, London, UK; 3 Division of Nutrition, St John's National Academy of Health Sciences, Bangalore, Karnataka, India; 4 School of Medicine, Dentistry & Nursing, University of Glasgow, Glasgow, UK; 5 Stable Isotope Biochemistry Laboratory, Scottish Universities Environmental Research Centre, East Kilbride, UK; 6 Department of Metabolism, Digestion and Reproduction, Imperial College London, London, UK; 7 Tropical Gastroenterology and Nutrition Group, University of Zambia School of Medicine, Lusaka, Zambia

**Keywords:** malnutrition, gastrointestinal function

## Abstract

Gut function remains largely underinvestigated in undernutrition, despite its critical role in essential nutrient digestion, absorption and assimilation. In areas of high enteropathogen burden, alterations in gut barrier function and subsequent inflammatory effects are observable but remain poorly characterised. Environmental enteropathy (EE)—a condition that affects both gut morphology and function and is characterised by blunted villi, inflammation and increased permeability—is thought to play a role in impaired linear growth (stunting) and severe acute malnutrition. However, the lack of tools to quantitatively characterise gut functional capacity has hampered both our understanding of gut pathogenesis in undernutrition and evaluation of gut-targeted therapies to accelerate nutritional recovery. Here we survey the technology landscape for potential solutions to improve assessment of gut function, focussing on devices that could be deployed at point-of-care in low-income and middle-income countries (LMICs). We assess the potential for technological innovation to assess gut morphology, function, barrier integrity and immune response in undernutrition, and highlight the approaches that are currently most suitable for deployment and development. This article focuses on EE and undernutrition in LMICs, but many of these technologies may also become useful in monitoring of other gut pathologies.

## Introduction

Undernutrition has been a part of human experience throughout history and still accounts for 45% of deaths in children under the age of 5.^1^ Intuitively, it would be expected that the major manifestations of undernutrition would respond to provision of nutrients, but this is not reliably true. Over the last decade, consistent evidence supports the surprising conclusion that one widespread manifestation of undernutrition, stunting (impaired linear growth in children^2^), is largely refractory to nutritional supplementation. Both systematic reviews and randomised controlled trials support this conclusion since only about one tenth of the growth deficit can be corrected by nutritional supplements alone.^3–6^ Children with wasting (low weight for height), a distinct but often overlapping manifestation of undernutrition, also face long-term health defects, which persist despite therapeutic refeeding. One hypothesis for the resistance of stunting and wasting to dietary interventions is that gut dysfunction compromises nutrient availability, uptake and use required for healthy growth.^7 8^ Subclinical gut dysfunction in the context of a marginal diet and chronic enteropathogen carriage, common in many low-income and middle-income countries (LMICs), is termed ‘environmental enteric dysfunction’ in view of geographical and clinical observations that suggest it is related to environmental conditions.^9 10^ The pathological basis of this dysfunction is best referred to as ‘environmental enteropathy’ (EE).^11^

**Figure 1:**
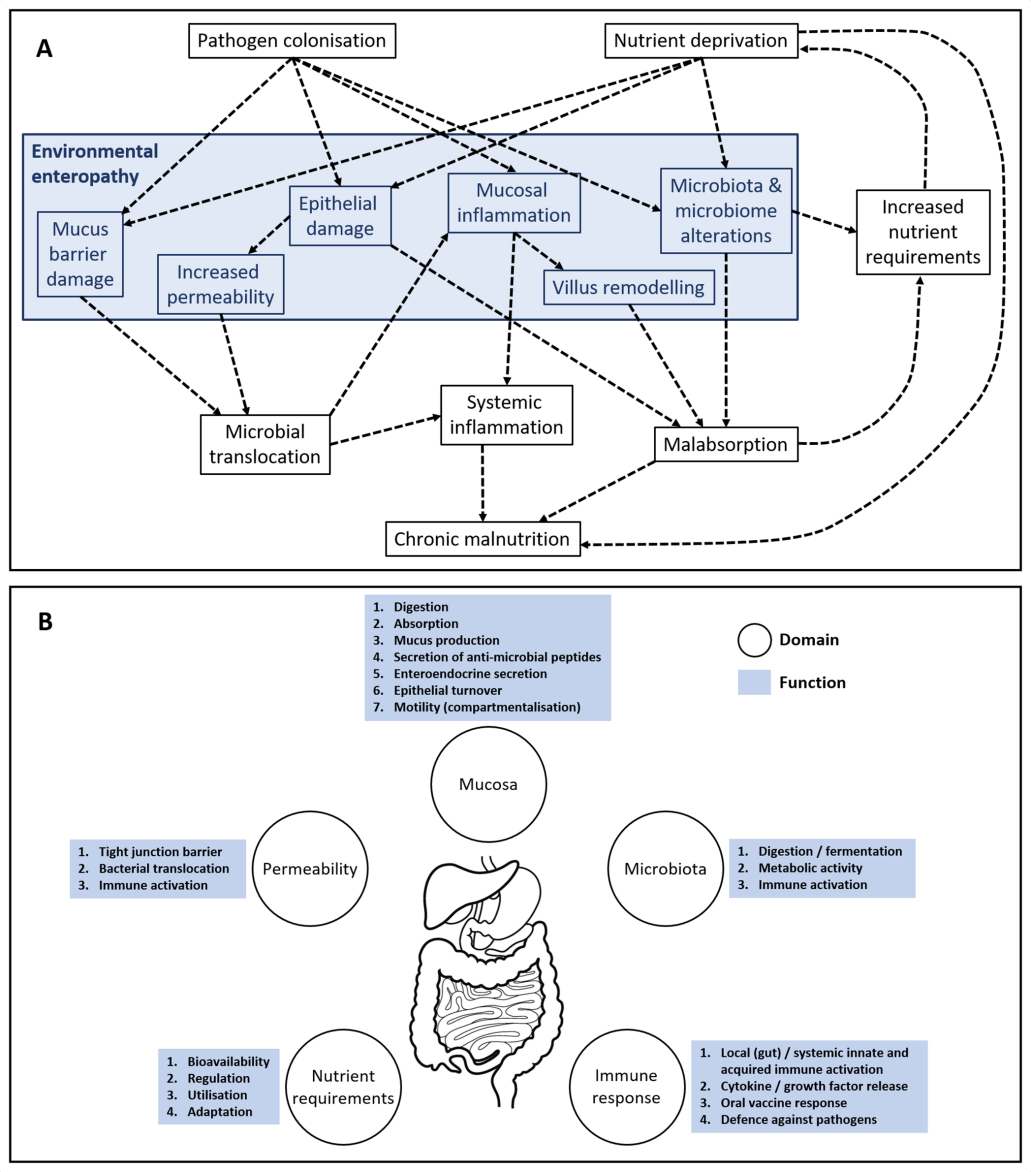
Pathology and domains of gut dysfunction. (A) Diagram illustrating the authors’ interpretation of the pathology of EE (blue boxes) showing links to broader features of gut dysfunction and undernutrition (black boxes). (B) Domains of gut function that appear to be aberrant in EE. Functional tests that report across each domain may shed new light on both the domains of dysfunction and the degree of dysfunction in various forms of undernutrition. EE, environmental enteropathy.

Investigation of EE has been limited hitherto by the need for invasive approaches, including small intestinal biopsy, and very few non-invasive tests are in widespread use. EE is characterised by multiple changes in the mucosa of the small intestine, though adults and children also have some changes in gastric and possibly colonic mucosa. Figure 1A illustrates the pathology of EE (as interpreted by the authors), which includes epithelial damage, leading to loss of barrier function with impaired secretory and absorptive function, accompanied by inflammation in the lamina propria.^12 13^ To what extent these pathological changes in the gut result in functional changes are yet to be fully understood. Figure 1B highlights the key domains and central functions thought to be impacted in EE. One important domain is gut permeability, which can be measured by assessing the urinary recovery of orally ingested sugar molecules using tests first developed in the 1960s and 1970s such as the lactulose:mannitol (L:M) test.^14 15^ However, the L:M test has several problems: there are multiple testing protocols leading to difficulties with comparisons between studies; it is time-consuming; and the assays rely on high-cost and specialised laboratory reagents and equipment that are often unavailable at (or near) the point-of-care (POC).^15^

Beyond permeability, other biomarkers have also been recognised to be associated with EE and undernutrition.^16 17^ Using molecular diagnostics, some of the most promising markers of EE pathophysiology are faecal and systemic peptide inflammatory biomarkers^18–22^ and growth hormones.^21 22^ However, most existing biomarkers provide only a proxy indicator of pathophysiology and immune cell function in undernutrition, and the mechanisms that link inflammation to stunting and wasting are poorly characterised.^23 24^ An emerging domain in EE is the gut microbiota. Previous work has reported that the gut microbiota follows a patterned progression of maturation during the first 2 years of life.^25^ Disturbance of this microbial succession, leading to microbiota immaturity, is associated with stunted growth and poor outcomes in therapeutic interventions for the most severe manifestation of wasting, severe acute malnutrition (SAM; weight-for-height Z-score <−3 and/or bilateral pitting oedema).^25^ An altered gut microbiota has also been reported in children displaying EE.^26^ Alterations to gut microbiota composition and function may influence uptake/use of nutrients and immune defences against enteropathogens, thereby contributing to poor childhood growth.^27^ However, at present, it is unclear if alterations in the gut microbiota drive gut dysfunction in malnutrition or are a consequence of aberrations in nutrient assimilation in the proximal gut.^28^ Lastly, assessment of digestive and absorptive functions has been largely neglected in EE, which is startling given the primary role of the small intestine in making essential nutrients from the diet available to the host.

Current techniques for the assessment of EE largely rely on technologies that require high costs, time, infrastructure and expertise. These limitations—combined with the resource-limited settings in which undernutrition is most prevalent—indicate that there is an urgent need for new and improved tests. Ideally, such tests should be easy to carry out, give reliable information without the need for specialised equipment/training, be significantly less costly than current approaches, generate timely results to guide clinical responses and be highly informative about specific pathophysiological domains. In this review, we evaluate a range of emerging technologies that may address these challenges and that are suitable for application to the investigation of undernutrition in LMICs. The focus is on systems that have the potential to be deployed at the POC (defined here as devices that are sufficiently simple and user-friendly to allow use in primary care settings, rural health facilities or even domestic environments), as these may permit large-scale, longitudinal research studies. Such technologies have utility beyond undernutrition and could provide valuable new insights into clinical gut pathologies such as IBD.

**Key message: the need for improved assessment of gut function**

- Undernutrition is underpinned by a multifaceted breakdown in gut function known as environmental enteropathy (EE).
- Current technologies for assessment of gut function are typically expensive, invasive and unsuitable for use at point-of-care (POC) in low-income and middle-income countries (LMICs), meaning that the role of the gut in undernutrition is poorly understood.
- New tools that are deployable at scale in POC settings would be hugely beneficial in improving understanding of EE and assessing the impact of interventions designed to improve clinical outcomes in undernutrition.

## Improving assessment of gut function

### Direct imaging and sampling of the gut

#### Endoscopic imaging capsules

**Figure 2:**
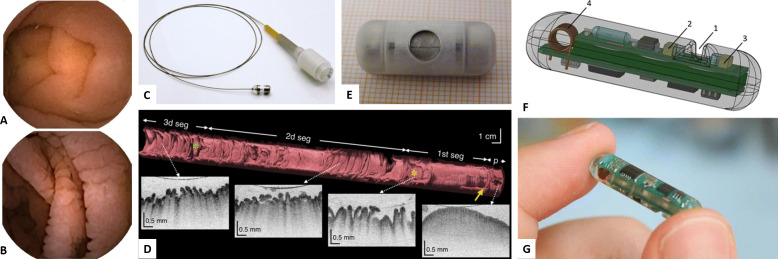
Capsule technologies for imaging, sensing and sampling. (A, B) Wireless capsule endoscopy images of the small intestine in (A) a healthy volunteer and (B) a patient with coeliac disease. Reprinted from Ciaccio *et al* 32, Copyright (2010), with permission from Elsevier. (C) Photograph of a TC-OCT system. Reprinted from Gora *et al* 35, Copyright (2018), with permission from Elsevier. (D) Example TC-OCT data collected in the small intestine demonstrating the capability to provide direct imaging of villi over large areas. Reprinted from Gora *et al* 35, Copyright (2018), with permission from Elsevier. (E) Photograph of a magnetically actuated wireless sampling capsule (shown on graph paper for scale). Republished with permission of The American Society of Mechanical Engineers, from Simi *et al* 46; permission conveyed through Copyright Clearance Center, Inc. (F, G) Model (F) and photograph (G) of the HemoPill sensing capsule for detection of intestinal bleeding. 1, recess in which blood is detected; 2, light detector; 3, light sources; 4, antenna for data transmission. Reprinted by permission from Springer Nature, from Schostek *et al* 51, Copyright (2016). TC-OCT, tethered capsule optical coherence tomography.

Beyond work undertaken at a limited number of specialised centres able to perform intestinal biopsies in children, relatively little is known about the structural changes in the gut mucosa in EE. In addition, due to ethical reasons and the limited access to intestinal tissue, there is an added challenge in defining normal gut morphology in a child, particularly at key stages of development, such as the introduction of complementary feeding. While numerous commercially available clinical imaging techniques exist that are suitable for in-hospital assessment of the morphology of the gut mucosa in EE (eg, endoscopy, confocal laser endomicroscopy, etc; see Thompson *et al* 29), such approaches are invasive and hence are unlikely to be deployable in LMICs outside of specialised centres. Capsule-based imaging technologies are less invasive and provide comparable imaging capabilities to traditional endoscopes and endomicroscopes. The most widely used is wireless capsule endoscopy,^30^ in which an untethered capsule (approxmately 10×30 mm) is swallowed by the patient and records video footage as it passes through the gastro-intestinal (GI) tract. While this approach provides slightly poorer quality images than standard endoscopy, it is considerably less invasive and safe use has been demonstrated in children as young as 10 months^31^ (though future use in younger children or larger infant cohorts would require further miniaturisation). Furthermore, in combination with appropriate image processing, wireless capsule endoscopy can identify villous damage (see figure 2A, B) with high sensitivity and specificity,^32^ indicating a potential applicability to EE and other clinical conditions where villous damage/remodelling is known to occur.

Higher-resolution imaging capsules also exist, and the most advanced (in terms of developmental progress) is tethered capsule optical coherence tomography (TC-OCT).^33^ Optical coherence tomography (OCT)^34^ is a structural imaging technology that provides depth resolved images at high speed and with resolutions of 10–20 µm (which allows direct imaging of individual villi). In TC-OCT, the imaging technology is contained within a capsule that is swallowed by the patient (figure 2C). This capsule is attached to a fibre-optic tether, which is used to deliver/collect light and to retract the capsule once it has descended to a desired point in the GI tract. During this pull-back procedure, thee-dimensional (3D) images are acquired at high speed: imaging of the entire oesophagus has been demonstrated within approximately 60 s.^33^ Furthermore, large-area imaging of the villous morphology in the small intestine has recently been demonstrated without sedation (see figure 2D),^35^ indicating that TC-OCT has potential applications in EE.

Finally, tethered capsule confocal laser endomicroscopy systems have also been reported (eg, see Tabatabaei *et al* and Kang *et al* 36 37), which provide higher resolutions than TC-OCT (approximately 1 µm) but with slower imaging speeds. These tethered capsule confocal endomicroscopes offer the opportunity to image morphological features of gut dysfunction such as crypt hyperplasia and lymphocyte infiltration, which is unlikely to be possible using alternative techniques.

#### Sampling/biopsy capsules

Beyond imaging morphological changes in the mucosa, minimally invasive sampling of luminal contents or biopsy capabilities would address several domains of EE. Direct assessment of mucosal injury would illuminate tissue and cellular changes in EE. Similarly, sampling of the luminal metabolome could identify changes in nutrient digestion and absorption, while sampling of the small intestinal microbiota would allow identification of microbial species present in the lumen and mucosa. However, endoscopic biopsy represents an expensive, invasive procedure that is unlikely to be ethically acceptable in longitudinal studies performed in infants. Carrying out this task using a capsule would be both less invasive and more likely to be achievable in longitudinal investigations.

Prior to the advent of endoscopy, the Crosby-Kugler capsule—a tethered capsule containing a syringe-driven knife—was the most common device for collection of tissue biopsies in the GI tract,^38^ and modified capsules were even developed for paediatric use (eg, see James^39^). However, use of Crosby-Kugler capsules has ceased due to the relative ease with which biopsies can be performed using modern endoscopes. Nonetheless, some minimally invasive sampling devices do still exist, with two tethered capsule systems having been commercially available over recent years: the Entero-Test (HDC diagnostics) and the Cytosponge (Covidien; eg, see Benaglia *et al* 40). These are both useful tools and, interestingly, the Entero-Test has been applied to studies that have clear relevance to undernutrition and EE: an investigation of Tropical Sprue^41^ and studies of the upper intestinal microbiota in malnourished children.^42 43^ Despite this, both suffer from the limitation that the samples can become contaminated during the withdrawal process as the sampling mechanism is not location specific.

Further devices are under development that aim to offer sample capture with location specificity. For example, BioMe Oxford (http://www.biome-oxford.com/) is developing an untethered capsule that is designed to collect samples from specific locations within the intestine while avoiding the aforementioned issues of contamination. In addition, the IntelliCap (Medimetrics)—a capsule originally designed for controlled drug release—may also provide the requisite location-specific sampling capabilities, and this device now has a CE mark for sampling of intestinal fluid^44^ (although its dimensions—11 mm diameter, 26 mm length—will preclude safe use in young children).

Finally, further research into the development of improved sampling capsules is still under way (eg, see the recent review of robotic endoscopic capsules by Ciuti *et al* 45), and one interesting example is the magnetically actuated untethered biopsy capsule reported by Simi *et al* 46 (see figure 2E). While such capsules are currently in the development phase, they illustrate the potential for wireless, untethered sample/biopsy collection. Thus, in the future, this area of research may well yield devices that are ideally suited to the sampling requirements in undernutrition.

#### Sensing and combination (multifunctional) capsules

A final topic of interest is the development of sensing capsules and ‘combination’ (multifunctional) capsules. While research in this area is somewhat limited, capsules with advanced capabilities for sensing, imaging and/or sampling would certainly be of use in phenotyping EE by capturing multiple domains of EE either in targeted (sensing) or untargeted (sampling) approaches.

In terms of sensing (ie, non-imaging) capsules, a small number have been reported, with some now in clinical use. Capsule sensing capabilities available clinically include pH, temperature, pressure and motility, as discussed in the recent review by Cummins *et al*.^47^ Gas sensing capsules have also been reported, which have recently been trialled in both animals^48^ and humans,^49^ and which may be well suited to investigation of the microbiota in undernutrition (both its composition and metabolic activity).

In addition, Ovesco Endoscopy AG recently brought to market an ingestible, untethered, sensing pill that provides diagnosis of GI bleeding. This device—known as the HemoPill (see figure 2F, G)—consists of a 6.5 mm (diameter)×25.5 mm (length) cylindrical capsule with an active sensing region capable of detecting the presence of blood.^50^ The sensing is based on optical spectroscopy (in particular, the relative transmission of light of two different colours through the sensing region), and the device has been validated in adult volunteers.^51^

While the detection of GI bleeding may not be of direct relevance to undernutrition, this demonstrates the potential to develop wireless sensing capsules for a range of targets. As an example of a potential application in undernutrition, a capsule similar to the HemoPill but with fluorescence sensing capabilities could allow minimally invasive assessment of gut permeability by detecting the leakage of intravenous fluorescent dyes into the intestine (in a similar manner to that reported by Kelly *et al* using confocal laser endomicroscopy^52^). Indeed, a wireless fluorescence imaging capsule was recently reported by Al-Rawhani *et al*,^53^ indicating that the technological development required for this purpose is not unrealistic.

Multifunctional capsules are even more rare than sensing devices. Nonetheless, capsules with multiple sensing capabilities exist—for example, the SmartPill (Medtronic)^54^—and such devices could be useful in undernutrition, with possible examples including a wireless capsule endoscope with fluorescence sensing capabilities (to provide localisation of intestinal leakage) and/or an imaging capsule with controllable sampling/biopsy capabilities (to permit sampling at known locations and correlation with quantification of villous morphology).

Overall, while capsule technologies provide numerous opportunities for characterising EE in situ with protocols that are less invasive than standard endoscopy, all are still likely to require quite advanced clinical facilities. Thus, they may help to reduce the invasiveness of undernutrition studies that take place within hospitals or major research centres, but are not likely to be suitable for POC use (in primary care centres, rural health facilities or domestic environments) in the near future. In addition, large-scale use in children/infants will require further miniaturisation. Nonetheless, capsule technologies are better suited for use in LMICs than traditional endoscopy/endomicroscopy, simply because they are less invasive and can also be less expensive.

### Next-generation measurements of gut permeability

Increased gut permeability is thought to be one of the hallmarks of EE, but assessment is still reliant on urine sugar permeability tests developed in the 1960s and 1970s.^55^ New techniques such as optical spectroscopy afford the opportunity for minimally invasive or non-invasive assessment of human tissue/samples. Specimens are illuminated with light, and the resulting signals are quantified as functions of wavelength (detailed descriptions can be found elsewhere; for example, see Lakowicz and Thompson and Yang^56 57^). While spectroscopy provides numerous clinical opportunities, it has previously been proposed that the most promising application of fluorescence (and reflectance) spectroscopy for undernutrition may be in the measurement of intestinal permeability.^29^ This approach would involve using transcutaneous fluorescence spectroscopy (ie, fluorescence measurements made through the skin) to assess the uptake of orally ingested contrast agents from the gut into the bloodstream. This technique is comparable in concept to previous studies performed in animals^58–60^ and is currently being validated in humans.^61^ Briefly, it involves patients drinking an oral dose of a fluorescent contrast agent (dye) and a wearable sensor or probe being used to detect and quantify the uptake of the contrast agent from the gut into the bloodstream. As the rate and degree of uptake are affected by changes in permeability, this approach will potentially allow rapid, non-invasive assessment of intestinal permeability, without the need to collect urine samples. As discussed previously,^29^ this method requires further validation but has the potential to improve on current techniques used to quantify permeability in terms of invasiveness, reliability and ease of deployment.

A further form of optical spectroscopy that may be useful in undernutrition is Raman spectroscopy^57 62 63^), which has been used in the detection and classification of bacteria^64–68^. While Raman signals are often weak in comparison to typical fluorescence or reflectance spectra (meaning that the detection systems are usually more advanced and expensive), several handheld Raman spectrometers are nonetheless available commercially at the time of writing (eg, CBEx, Snowy Range Instruments, USA; http://www.wysri.com/cbex/), indicating potential utility for POC assessment of bacterial translocation and/or the microbiota in undernutrition. However, to fully realise this potential in a POC application, improved sample preparation techniques will be required that can provide sufficient signal-to-noise ratios without the need for time-consuming, laboratory-based preprocessing (eg, similar to the protocol recently reported by Boardman *et al* for detection of bacteria in blood samples^69^). In addition, for analysis of the microbiota, innovative sample collection techniques (eg, see Sampling/biopsy capsules section) may also be beneficial. Interestingly, Raman spectroscopy has also been used for analysis of breath samples (see further details in Tracing nutrient digestion and absorption non-invasively section), indicating that a number of potential development routes exist for application to undernutrition.

### ‘Omics’ picture: microbiome and metabolome

#### Microbiomics

The pathology of EE is driven, in part, by infection and subclinical pathogen carriage.^70^ Large, multicountry studies have reported that enteropathogen carriage is almost ubiquitous among children in LMICs^20 71^ and is associated with increased intestinal permeability, enteric inflammation and faltered growth.^72^ In addition to pathogenic micro-organisms, emerging evidence suggests an influential role of the commensal microbiota—and the importance of its genetic make-up (often termed the microbiome)—in both EE and child undernutrition.^26 27^ Advances in sequencing technologies have facilitated assessments of microbial diversity in EE.

The rapid development of sequencing technologies has allowed for increased use of culture-independent clinical diagnostics of infectious diseases.^73^ So-called next-generation sequencing (NGS) technologies—including Roche 454 and Illumina platforms (MiSeq, HiSeq, etc)—have been widely employed for high-throughput analysis of mixed metagenomes, particularly those within the GI tract. However, the considerable size, time, cost and data output associated with these sequencing approaches render them largely inappropriate as POC technologies and have necessitated cold-chain shipping of samples to sequencing sites outside LMICs.

**Figure 3:**
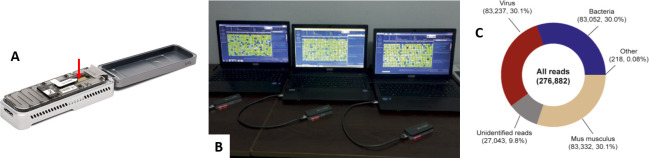
Portable sequencing using the MinION (Oxford Nanopore Technologies). (A) Photograph of the MinION handheld sequencing tool developed by Oxford Nanopore Technologies (source: https://nanoporetech.com/products/minion). Red arrow indicates where liquid samples are deposited for analysis. (B) Photograph of multiple MinION systems deployed for sequencing of Ebola. Reprinted by permission from Springer Nature, from Quick *et al* 74, Copyright (2016). (C) Example MinION data collected from a mixture of bacterial, viral and mammalian tissue (*Mus musculus*) material onboard the International Space Station demonstrating the suitability for remote deployment. Reproduced from Castro-Wallace *et al* 76 under the terms of the Creative Commons CC BY license.

To overcome these limitations, Oxford Nanopore Technologies has developed the MinION platform (figure 3A): a handheld sequencer that can generate gigabases of long reads (as opposed to short reads offered by other NGS platforms). The MinION can be powered through a laptop USB port and has significantly cheaper start-up costs (approximately $1000) than traditional sequencing platforms. ‘Real-time’ data generation also establishes the MinION as an attractive clinical diagnostics tool which could plausibly identify infectious agents within hours (vs days using traditional NGS platforms or culture techniques). Recently, the MinION was successfully used for surveillance of an Ebola outbreak in Guinea (figure 3B), with results generated within 24 hours of sample collection and sequencing times of 15–60 min.^74^ The platform has also successfully been used in Antarctica^75^ and on the International Space Station (figure 3C),^76^ demonstrating its ease of use in remote settings. The MinION has also recently been trialled for rapid sequencing of the faecal microbiome in preterm infants at increased risk of sepsis and necrotising enterocolitis, producing results comparable with Illumina MiSeq within 5 hours of sample collection.^77^ This approach was also able to rapidly assess antimicrobial resistance gene carriage within the microbiome, which poses great potential for clinicians to provide targeted antibiotic treatments, an issue that is particularly relevant in the context of SAM.

Despite the promise of the MinION, it is still limited by multiple factors when compared against traditional NGS methods (including coverage depth, error rates and sensitivity).^78 79^ Furthermore, POC deployment is dependent on DNA extraction and library preparation prior to sequencing. However, a new compact Oxford Nanopore platform (VolTRAX) is under development to automate library preparation directly from sample input.

Consistent data are now confirming that enteropathogens have a key influence in undernutrition, yet information on the role of the microbiota and microbiome remains sparse. Hence, portable sequencing technologies such as the MinION (and the SmidgION, a more compact sequencer designed to connect to a mobile phone) offer opportunities to improve understanding of the association between the microbiome and undernutrition through cost-effective and time-effective data collection in POC settings. Where there remains a significant challenge is in characterising the functions of the resident microbiota and identifying whether functional niches in the microbial community become impaired over time and with increased severity of EE.

#### Paper strip metabolomics

The rich data provided by metabolic profiling offer numerous potential opportunities in undernutrition. One solution to combining this with POC diagnostics is to develop paper chromatography methods of collecting and/or analysing samples in the field. Dried blood, urine or faecal spots deposited on paper substrates, combined with reversed-phase ultraperformance liquid chromatography and mass spectrometry (MS), have been widely investigated with varying opinions as to whether the metabolite and sensitivity losses encountered are acceptable with respect to diagnosis of specific pathologies. The advantage of dried blood spots or urine strips is that samples can be mailed to analysis centres or analysed on site if a suitable instrument is present. This enables patients to collect samples at home and can minimise stress to infants and children.

The use of metabolic profiling of dried blood spots in newborns was explored by several groups in the 1990s (eg, see Parker and Cubitt^80^). More recently, an untargeted metabolic profiling method based on liquid chromatography high-resolution MS was applied to newborn dried blood spots and was able to define characteristic profiles associated with ethnicity and birth weight.^81^

Another recent study compared fresh plasma and urine against dried spots/strips using multiple MS approaches.^82^ This study reported that paper substrates had only a small impact on the overall MS signals and that dried urine spots were inherently more stable than dried blood spots (with the majority of compounds in urine remaining stable at room temperature for at least 1 week postcollection). Other papers, however, have reported reasonable recovery of metabolic information from blood spots but have suggested a loss of compounds from urine spots using negative ionisation (eg, see Michopoulos *et al* 83).

Long-term storage (over a 15-year period) has also been evaluated by comparing dried blood spots stored at room temperature against serum stored at −80°C, demonstrating degradation of most components in the dried blood spots.^84^ Hernandes *et al* 85 reviewed sample handling and storage procedures for dried blood spots and noted the importance of standardising paper substrates, drying times and storage temperatures, with the recommendation that the dried blood spot cards are stored at −20°C or lower.

In summary, paper strip metabolomics can be used to overcome some of the problems of obtaining high-quality samples at POC and is attractive because it is reagent free and relatively inexpensive. However, a proportion of metabolites are lost, meaning that the utility of this method is dependent on the nature of the specific metabolites of interest.

#### Miniaturised chromatography and mass spectrometry

There is also a drive towards development of miniaturised chromatographic and direct injection MS systems from the perspectives of both handheld POC diagnostics and green (sustainable) chemistry. Efforts to miniaturise high-performance liquid chromatography (HPLC)—for example, to substantially reduce column diameter^86^ or to adopt microfluidic technology (www.anywhereHPLC.co.uk)—have resulted in some success, although the technology is currently not widely available.

Microchip electrophoresis has been developed in a miniaturised, portable format that lends itself to clinical diagnostics (eg, see Guihen, Qian *et al* and Kamruzzaman *et al* 87–89). However, compared with traditional metabolomics assays, the microfluidic electrophoresis has generally focused on a small number of analytes. Ambient ionisation MS was developed in 2004 and enables the user to operate without sample preparation or chromatographic separation. This has since been applied to newborn screening and monitoring of therapeutic drugs.^90^

Overall, technology advances in direct injection and ambient MS promise to facilitate the development of POC mass spectrometers, which are portable, require low sample volumes and are relatively cheap to run. These would be of value for metabolic assessments as well as for other domains of gut dysfunction—for example, as breath analysers and for proteomics analysis of immune biomarkers (see further details in Tracing nutrient digestion and absorption non-invasively section and Gut-associated immune function and inflammation section). Various prototype platforms exist, however, considerable further development (including further miniaturisation and cost reduction) is required before these will be widely deployed in clinical settings in LMICs.

### Tracing nutrient digestion and absorption non-invasively

A limited number of recent studies have successfully employed stable isotope techniques (involving assessment of blood samples on mass spectrometry platforms) to measure digestion and absorption of amino acids in both children and adults in LMICs (eg, see Shivakumar *et al* and Kashyap *et al* 91 92). Despite this, there is still surprisingly little information in malnutrition on the impact of gut dysfunction on the ability to process essential dietary nutrients to make them available to the host. Moreover, it remains unclear whether undernutrition, EE and enteropathogen infection overlap to increase requirements for essential nutrients as is observed in periods of high nutrient requirements.^93^

Digestion and absorptive capacity can be assessed using both metabolomic and targeted approaches, and breath is an attractive sampling pool for such measurements because it can be sampled non-invasively in children. Breath tests can be categorised into untargeted analysis for the characterisation of volatile organic compounds (VOCs) or targeted analysis on a single or limited number of gaseous species. Within targeted breath analysis, an additional functional dimension is accessed through the use of a stable isotope tracer. By sampling the isotope excretion in a breath metabolic end product, the targeted test assesses the functional capacity of a metabolic pathway or digestive/absorptive capacity.

**Figure 4:**
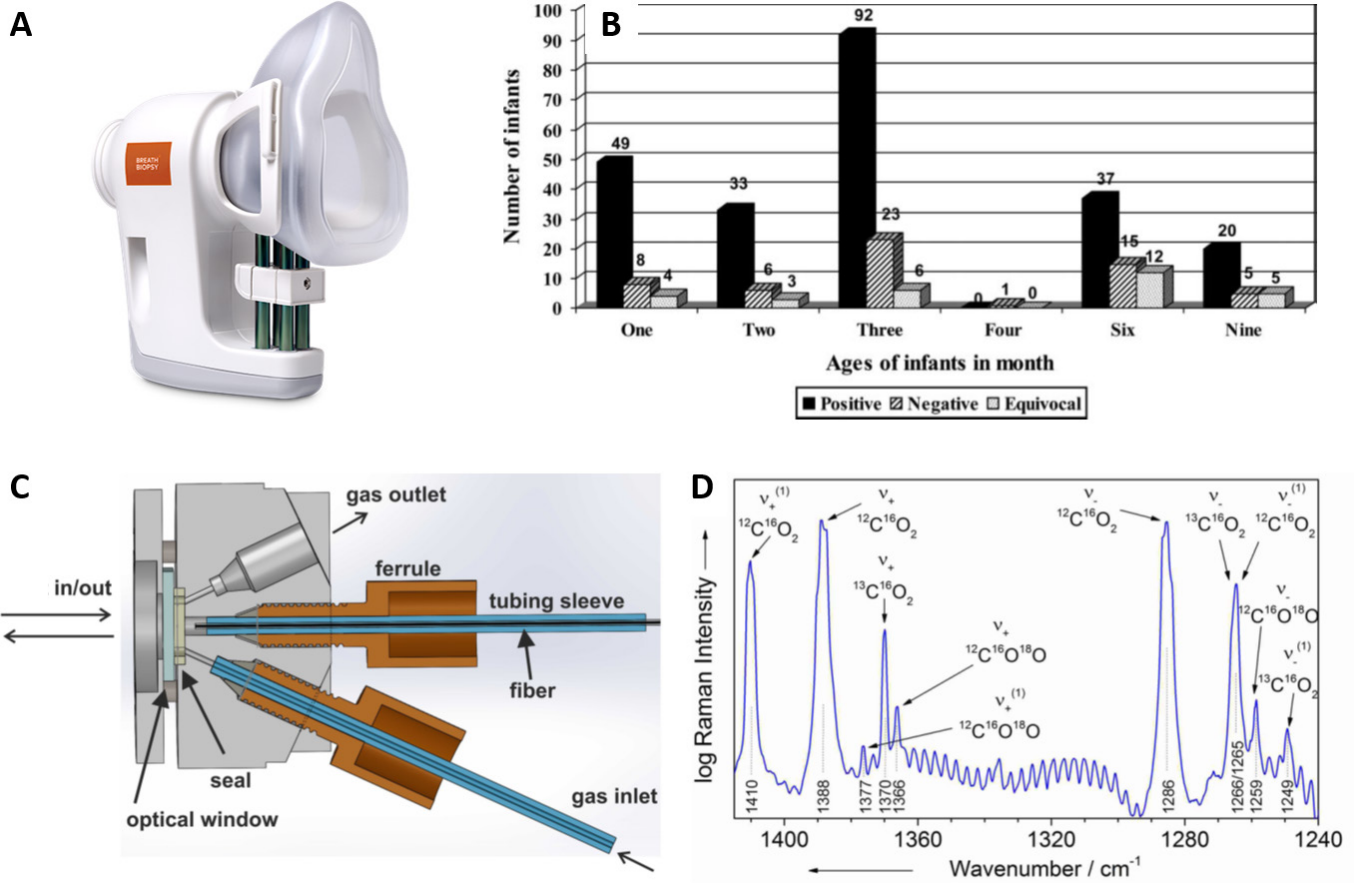
Point-of-care breath tests. (A) A portable breath sample collection tool (ReCIVA breath sampler, Owlstone Medical) for use in conjunction with the breath biopsy central analysis facility (source: https://www.owlstonemedical.com/products/reciva/). (B) *Helicobacter pylori* infection data collected using a non-invasive ^13^C urea breath test in Pakistani infants demonstrating the suitability of breath tests for use in large cohorts. Reprinted from Nizami *et al* 97 with permission from Wolters Kluwer Health, Inc. The Creative Commons license does not apply to this content (figure 4B). Use of the material in any format is prohibited without written permission from the publisher, Wolters Kluwer Health, Inc. Please contact permissions@lww.com for further information. (C) Diagram of a compact and alignment-free gas sensing system based on FERS. Reprinted with permission from Hanf *et al*.^109^ Copyright (2014) American Chemical Society. (D) Example FERS data demonstrating the capability to detect and identify ^13^C at low concentrations. Due to the linear relationship between the FERS signal intensity and gas pressure (and because different isotopes exhibit spectral peaks at distinct frequencies), FERS spectra can be interpreted to extract concentrations of desired gaseous species (eg, ^13^C) simply through quantification of the intensity of specific peaks in the spectra (assuming an appropriate calibration procedure has been performed). Reprinted with permission from Hanf *et al*.^109^ Copyright (2014) American Chemical Society. FERS, fibre-enhanced Raman spectroscopy.

Untargeted breath analysis is an established tool in investigating pulmonary inflammatory disease and primarily focuses on breath condensates and VOCs that can be trapped on absorbent materials. The mainstay analytical technology is mass spectrometry, which requires dedicated centralised facilities (reviewed in Tang *et al* 94); however, recent efforts to miniaturise gas chromatography-mass spectrometry (GCMS) have been reported^95^ (see previous discussion). Thus, much of the focus in the field has been on gas sample collection and fidelity in storage prior to analysis at a central facility such as the Breath Biopsy (eg, see figure 4A). Advances such as wafer technology for entrapment of selective species by field asymmetric ion mobility mass spectrometry (Owlstone Medical) and electronic nose technologies (Sensigent) are attractive, but the lack of robust information on which breath biomarkers to use in EE is a current limitation.

Targeted breath analysis using stable isotopes to probe the function of a metabolic, bacterial or xenobiotic pathway is attractive in undernutrition because of its potential sensitivity and specificity and its relevance to numerous domains of gut dysfunction.^96^ This paradigm was established by the ^13^C urea breath test, which has been used extensively to map Helicobacter pylori infection at population scales (figure 4B).^97^ The simplicity of the test—administration of a small amount of ^13^C urea and collection of one or two samples of breath CO_2_ within 30 min—is highly attractive in a test demonstrating very high sensitivity and specificity,^98 99^ allowing prompt clinical decision making.

Stable isotope breath tests have also been applied to numerous studies relevant to EE and undernutrition. To assess the availability of dietary nutrients to meet nutritional requirements, the indicator amino acid oxidation technique has been deployed, demonstrating similar lysine requirements in Western^100^ and Indian children.^101^ To what extent these requirements are altered by EE, malnutrition or entropathogens in children is largely unknown, although evidence from undernourished men suggests an increased requirement for indispensable amino acids.^102 103^ ^13^C-trigylceride digestion was shown to be variable in Jamaican children on admission to hospital and through recovery from SAM.^104^ In the Gambia, assessment of ^13^C-starch digestion in weaning children showed that those children with weight-for-height Z-scores of less than zero benefited most when supplemented with amylase-rich flour-treated feeds, which suggests impaired starch digestion capacity related to both age and weight.^105^ Thus, opportunities for the use of breath tests in the study of EE are widespread, but their deployment has been limited to date by resource and technical challenges.

The historical reliance on expensive isotope ratio MS measurements for breath ^13^CO_2_,^106^ coupled with the high technical expertise required to operate such instrumentation, has led largely to the use of centralised facilities for breath ^13^C analysis. Nonetheless, the development of non-dispersive infrared (IR) spectrometry for the assessment of ^13^C enrichment in CO_2_ 107 yielded the first site-deployable IR isotope systems (eg, Kibion IRIS, Exalenz Breath ID, UBiT-IR300, Heli-FAN/FAN-HP and HEADWAY HCBT-01), which have been used in *H. pylori* community screening programmes in LMICs.^108^ More recent IR laser-based systems have extended analysis capability to permit assessment of larger sample numbers (eg, ThermoFisher Scientific Delta Ray, Mirico, Picarro and Los Gatos Research). In addition, Raman spectroscopy has also been investigated as a tool for rapid analysis of breath samples. For example, Hanf *et al* described fibre-enhanced Raman spectroscopic analysis of exhaled human breath for quantitation of ^13^CO_2_ and other breath biomarkers (figure 4C, D).^109^ Further development is still required. However, given the attractiveness of breath sampling for diagnostic purposes and the pace of technological innovation, it is highly likely that handheld breath tests for VOCs and isotope-labelled target metabolites are on the horizon.

### Gut-associated immune function and inflammation

Elevated systemic and intestinal inflammatory biomarkers are associated with mortality among children with SAM^110 111^ and linear growth defects among children growing up in LMICs.^19 22^ Immune function in the gut can be inferred indirectly from soluble mediator levels in tissue-specific (eg, stool, saliva, biopsy) or systemic (blood) samples, but these cannot be linked to cellular source or stimulus, limiting their utility for understanding pathogenesis.^24 112^ Furthermore, direct assessment of cellular immune function has been extremely limited in studies of undernutrition in LMICs to date.^23 112^

Significant advances in new technologies for immunology research offer potential to resolve tissue composition and function of tissues at a single-cell level (eg, single cell ‘omics’,^113^ multiphoton microscopy^114^ and 3D tissue culture^115^). For example, single-cell transcriptional analysis of gut biopsy specimens from children with Crohn’s disease versus healthy children reveals unique epithelial cell profiles associated with inflammation.^116^ Increased understanding of pleiotropy and redundancy within networks of immune cells and mediators has also led to adaptation of traditional immunology assays to simultaneously assess multiple facets of the immune response rather than one biomarker at a time (termed ‘multiplexing’). For example, mass and spectral cytometry allow simultaneous detection of >100 cellular markers compared with <30 markers via traditional flow cytometry.^117 118^ However, very few of these advances have been translated to undernutrition/EE research, and the size, cost and complexity of the associated detection hardware are a significant barrier to translating cutting-edge cell-immunology and tissue-immunology methods beyond high-income laboratory settings. Notwithstanding the potential for these promising methods, we focus further on technological advances/adaptations that are closer to POC/near-POC assessment of gut-associated immune function and inflammation in LMICs.

#### Current assays evaluated in cohort studies

Few inflammation assays and no immune function assays currently meet WHO criteria for suitable POC tests for resource-limited settings.^119^ However, a range of basic laboratory technologies have been used to characterise immune mediators in undernutrition, including ELISA,^19 20 110 120–122^ quantitative PCR and microarray,^123 124^ GCMS,^110 111^ transcriptomics^125^ and flow cytometry.^24 112 126^ These approaches are routine for assaying a range of biomarkers in high-income laboratory settings and, in some cases, provide timely results (hours–days) to inform clinical management. However, they remain underused in adequately powered cohort studies of undernutrition/EE.

Organoid tissue culture techniques developed over the past decade use primary stem cells to generate self-assembling 3D mini-organs in vitro that more accurately recapitulate the organ environments than traditional cell culture. Organoids generated from gut tissue (enteroids or mini-guts) are beginning to be used to model EE (eg, Study of EE and Malnutrition in Pakistan^127^). Informed by transcriptomic studies using duodenal biopsy specimens from young children living in rural Pakistan, such organoid systems have shown that gene signatures of mucosal leucocyte activation (among other pathways) are associated with wasting.^125^ Enteroids would offer a way to explore gut leucocyte dynamics in vitro but can take days to weeks to establish, are subject to high interindividual variability and require tissue culture facilities that may not be available at POC in some settings.^128^

#### Emerging approaches

Numerous alternative techniques/approaches are emerging that may be better suited to POC assessment of immune function and inflammation. *Lateral flow immunoassays (LFIA)* are based on membrane strips that take up liquid samples by capillary action and detect the presence/absence of specific soluble proteins via recognition elements, usually antibodies, embedded in spatially defined zones. LFIA—already widely used for other conditions—have equivalent sensitivity and specificity to ELISA but faster turnaround (minutes vs hours–days) and fewer processing requirements. An LFIA-based C reactive protein (CRP) test has been validated as an indicator of acute-phase response to infections in SAM.^122^ LFIA can be adapted for simultaneous detection of multiple biomarkers via separation of detection sites, arraying membrane strips or using reporters with distinct signals.^129^ Multiplex LFIAs are beginning to be used to enhance diagnostics (eg, CRP and alpha-defensin in periprosthetic joint infection^130^) and could be readily adapted for EE (eg, myeloperoxidase, alpha-1-antitrypsin and neopterin are often assessed together as a combined marker of EE^19^ but currently rely on separate ELISA). Development of undernutrition-relevant LFIA has been limited by a lack of immune biomarker discovery studies.^24 112^

*Reverse transcription-based loop mediated isothermal amplification (RT-LAMP)* uses a series of strand displacement steps to allow continuous synthesis of DNA at a constant temperature.^131^ In combination with development of low-cost folded-paper microfluidics devices for sample preparation, electricity-free heaters^132 133^ and LFIA,^131^ these assays can be used to detect RNA/DNA biomarkers without the need for mains electricity, thermocyclers or high-cost detectors required for traditional PCR. RT-LAMP has been trialled for infection diagnosis in LMICs (eg, malaria in whole blood^132^ and HIV-1 in plasma^133^) and would also be suitable for immune mediators. Few RT-LAMP assays are commercially available and those that are have been reported to perform less consistently than PCR.^134^

**Figure 5:**
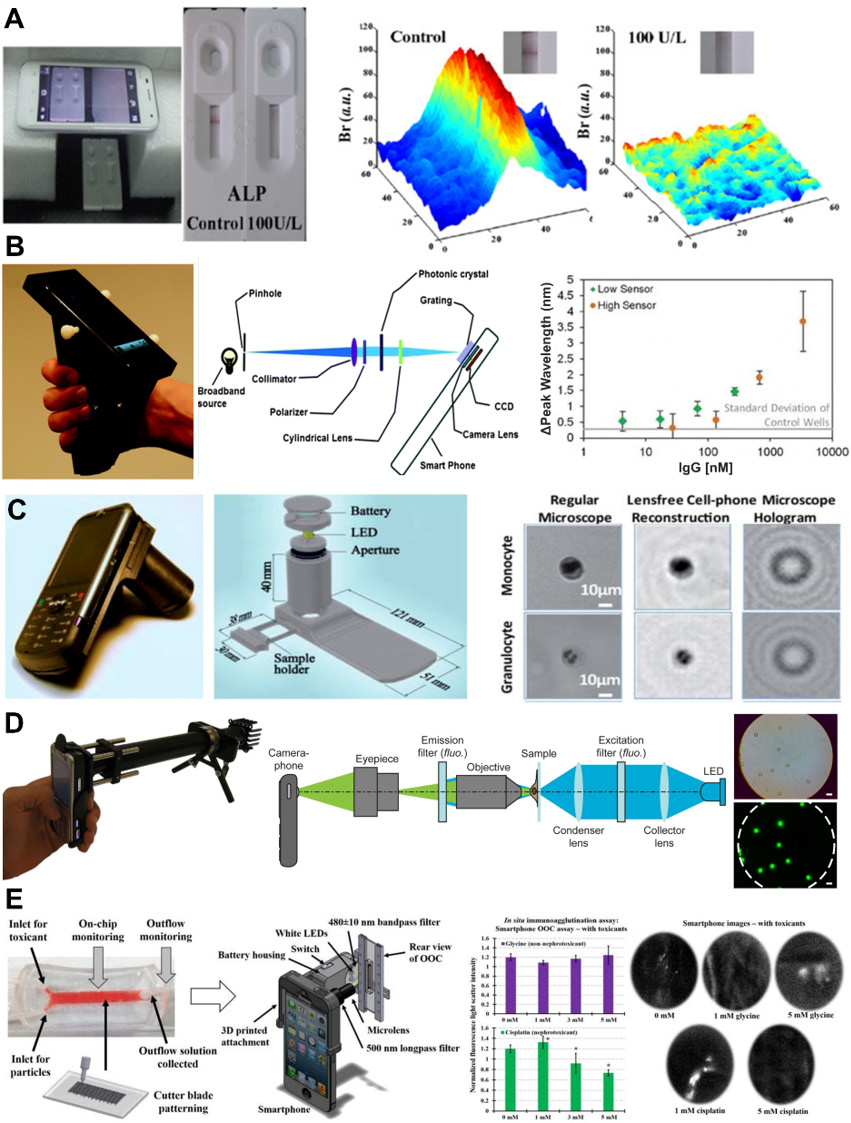
Smartphone-based detectors suitable for assessment of immune function (and other biomarkers) at point of care. (A) Image of smartphone-based reader for LFIA strip-based rapid tests for ALP activity in milk. No ALP is indicated by a visible band, ALP+ samples are indicated by the absence of a band (left). Br of bands is quantified on smartphone images using MATLAB (right). Adapted from Yu *et al* 139, Copyright (2015), with permission from Elsevier. (B) Image of a smartphone adapted for use as a spectrometer using a photonic crystal biosensor (left). Schematic of the optical components of the detector (centre). Example of label-free detection of changes in peak wavelength caused by the binding of a range of porcine IgG concentrations to an immobilised layer of protein (right). Low IgG concentration was detected by one photonic crystal sensor (green, low sensor) and high IgG concentration by a separate sensor (orange, high sensor). Adapted with permission from Gallegos *et al* 141, Copyright (2013), Royal Society of Chemistry. (C) Image (left) and schematic (centre) of a smartphone-based light microscope, which could be used for differential cell counts or label-free CD4+ T-cell detection. Comparative examples of immune cells imaged using a standard light microscope versus the smartphone device (right). Adapted with permission from Tseng *et al* 143, Copyright (2010), Royal Society of Chemistry. (D) Image (left) and schematic (centre) of a smartphone adapted for use as a fluorescence microscope, which has been trialled for detection of *Mycobacterium tuberculosis*-infected sputum samples (example of labelled beads shown on the right). Adapted from Breslauer *et al* 144 underthe terms of the Creative Commons CC BY license. (E) Example of how a lab-on-a-chip functional assay can be combined with a smartphone-based detector for point-of-care immune function assays. A schematic of the combined device (left: in this example, the lab-on-a-chip is lined with a renal adenocarcinoma cell line for assays of kidney function) and readouts from two functional assays using the smartphone-based detector (right: quantification of agglutination in response to a nephrotoxin and fluorescent imaging of cellular responses to the same toxin). Adapted from Cho *et al* 150, Copyright (2016), with permission from Elsevier. ALP, alkaline phosphatase; Br, brightness; CCD, charge-coupled device; LED, light-emitting diode; LFIA, lateral flow immunoassay.

*Smartphone-based detectors* use a combination of a light source, high-quality camera and customisable software within a smartphone to generate a range of low-cost, portable immunoassay detection platforms (reviewed in Liu *et al* and Geng *et al* 135 136). Such detectors would be ideal for LMICs, which can be well served by mobile phone networks despite limited laboratory resources in some settings. Smartphone-based detectors have been validated across a range of specimen types in small-scale studies for one-sample-at-a-time cuvette-based ELISA,^137^ 3D-printed multiplex ELISA^138^ and LFIA (figure 5A),^139^ with sensitivity of the latter significantly improved by combination with an image processing app.^140^ Smartphone-based detectors are also suitable for ‘label-free’ biomarker detection methods, which directly detect the molecule of interest via electrical, mechanical and/or optical transduction (eg, wavelength shifts induced by antibody binding to immobilised proteins^141^; figure 5B) rather than addition of a detection reagent.^142^ Addition of simple hardware can also be used for light and fluorescence microscopy (figure 5C, D).^143 144^ Though currently low-throughput, smartphone-based detectors could circumvent the size, cost and portability limitations of existing immunoassay detectors.

*Portable flow cytometers with automated analysis* would bring multiparameter cellular immunology to the bedside. Miniaturisation of detectors and fluidics systems has allowed for straightforward portable cytometers to be generated at low cost.^145^ A <US$5 mobile phone accessory has recently been developed in tandem with a disposable microfluidic device to count CD4+ T cells in <30 µL blood in <30 min without the need for fluorescent labelling, with results comparable to laboratory-based flow cytometers.^146^ Suitability for low-volume samples is desirable for studies involving sample collection from undernourished children. Existing prototypes require adaptation for multiparameter detection, high-throughput analysis and assay standardisation for POC use. In addition to detectors, standardisation of flow cytometry workflows for cell and tissue samples, which can vary widely, is necessary for consistent immunoassays across multiple sites and for longitudinal studies (eg, EuroFlow workflows for leukaemia immunobiology^147^).

Finally, *lab-on-a-chip assays* are miniaturised (millimetre to centimetre) microfluidics circuits that integrate one or more laboratory tests. They are specifically developed for POC use, are relatively low-cost, minimise cell handling and assay-to-assay variability, and reduce sample volumes required per assay.^148 149^ Some lab-on-a-chip devices have been developed in parallel with smartphone-based detectors^136 150^ (figure 5E). Commercial labs-on-a-chip are available for functional analysis of individual cell types (eg, neutrophil chemotaxis^151^) and could be used to validate and advance observations made in undernutrition using traditional cell culture.^23^ Based on organoid techniques, miniaturised models are being developed for whole tissues/organs (organ-on-a-chip^152^), including the gut.^153^ Gut-on-a-chip models of undernutrition/EE offer the potential for assessment of mucosal immune cell function in a standardised POC format.^128^ Lab-on-a-chip has been validated in chronic diseases in high-income settings.^152^ Gut-on-a-chip populated with epithelial cell lines (eg, Caco-2) are commercially available but yet to be validated in LMICs.^153^

**Key message: technologies for advanced assessment of gut function**

- A wide range of technologies exist that may facilitate point-of-care quantification of gut function in undernutrition.
- These include capsules for imaging and/or sampling of the gut, spectroscopic tools for non-invasive assessment of gut permeability, smartphone-based sensors for measurement of immune function, handheld sequencing devices and many others.

## Discussion

Undernutrition is underpinned by multifaceted alterations in the functional capacity of the gut, a condition often referred to as EE. Although many human conditions and disease states have manifest impacts on gut function, surprisingly little characterisation of gut function has been performed in undernutrition (beyond a small subsample of endoscopic investigations and a handful of biomarkers that are proxies for intestinal damage). Technological advances are potentially able to provide a more holistic view of different domains of gut dysfunction. Crucially, in EE, greater insight into gut dysfunction would help guide interventions aimed at restoring function and limiting the burden of undernutrition in LMICs.

The developing gut in infancy and later childhood is a highly dynamic organ and greater insight will undoubtedly come from minimally invasive methods to assess gut function that can facilitate repeated sampling through key development stages. Existing and emerging biomarkers from studies in LMICs (where stunting related to malnutrition, EE and enteropathogen burden is prevalent) highlight the complexity of the underlying pathophysiology of stunting. Therefore, it may be an unreasonable prospect to expect that a single test or biomarker captures this complexity. Crucially, however, deploying multiple new tests that can interrogate numerous complementary aspects of gut function simultaneously may help to improve understanding of EE and undernutrition. Here, we have outlined technological advances that can overcome the challenges in undertaking research to comprehensively interrogate gut function in children and adults with EE in LMICs (in both static and longitudinal manners). By using such technologies to improve understanding of EE (and gut function more broadly) and to monitor responses to interventions, future opportunities may also arise to better design interventions and even to identify children most at risk of stunting, thereby allowing targeted interventions.

A broad range of technologies for assessment of gut function are discussed above, and all have relative merits in the investigation of EE. The most promising approaches are summarised in table 1, which assesses each technique in terms of the technological readiness level (TRL; based on the scale originally devised by NASA^154^ with nine representing a mature, commercially available technology and one representing an initial concept), cost, potential mode of deployment, information provided, method for interpretation of results, suitability for use in LMICs and further development needed. Further detail is provided below to explain the potential areas of application, the advances that each technique may offer and the development required to facilitate use in LMICs.

**Table 1:** Important technologies for POC assessment of undernutrition

Technology	TRL	Cost (approx.)	Information provided	Mode of deployment	Interpretation of results	Suitability for LMICs (1–5)	Development required
Capsule systems							
Wireless capsule endoscopy	9	$$$	Villous morphology (indirect)	IH	Manual interpretation by specialist, automated image analysis feasible	2	–
Tethered capsule OCT	8	$$$	Villous morphology (direct)	IH/POC	Manual interpretation by specialist (training required), automated analysis feasible	3	POC validation
Sampling/biopsy capsules	5	$$$	Microbiota, biomarker quantification, metabolic profiling, villous morphology (via histopathology)	IH (sample freezing and shipping required)	Laboratory analysis required (eg, pathology, MS, etc)	2	Validation of location specific sampling
Optical spectroscopy							
Transcutaneous fluorescence spectroscopy	5	$	Permeability	POC	Automated, on-sensor analysis	5	Deployable device development, human validation
Raman spectroscopy	4	$$	Translocation, microbiota, breath sample analysis	POC	Automated analysis feasible (algorithm development required)	3	Sample preparation techniques, device development
Portable sequencing							
MinION	7	$$	Microbiota/microbiome, biomarker quantification, metabolic profiling	POC	On-site (POC) analysis using laptop	3	Sample preparation techniques, POC validation
SmidgION	6	$$	Microbiota/microbiome, biomarker quantification, metabolic profiling	POC	On-site (POC) analysis using laptop or smartphone	4	Sample preparation techniques, POC validation
Breath tests							
Untargeted	9	$$	Microbiota, biomarker quantification, metabolic profiling	POC (sample storage and shipping required)	Laboratory analysis required	3	Validation of biomarkers and sample stability, sample storage technology
Targeted	8	$$	Microbiota, biomarker quantification, metabolic profiling	POC	Laboratory analysis required	5	Devices for POC analysis, identification and validation of biomarkers
Immune function							
Smartphone-based ELISA/LFIA	6	$	Inflammatory biomarker quantification, immune function (indirect)	POC	Automated, on-sensor analysis	5	Validation for EE biomarkers, device/system optimisation
Miniaturised metabolomics							
Paper strip metabolomics	8	$	Biomarker quantification, metabolic profiling	POC (sample shipping required)	Laboratory analysis required	4	Validation of biomarker/metabolite stability
Portable mass spectrometry	3	$$	Biomarker quantification, metabolic profiling	POC	Automated, on-site analysis feasible (significant development required)	4	Device/system development

The most promising technologies discussed in this article are highlighted here. They are compared against one another in terms of their TRL, cost, the information that they provide, their mode of deployment, the way in which results are interpreted, their suitability for use in LMICs and the further development required. TRL is ranked on a scale of 1–9 (9 represents a mature, commercially available technology; 1 represents an initial concept). Mode of deployment is classed as either IH or POC, with POC referring to use in primary care settings, rural health facilities or domestic environments. Where necessary, the need to freeze and/or ship samples for analysis is also noted. As costs vary according to location and as some technologies have not yet reached market status, costs are provided on a relative scale only (ie, $, $$ or $$$). The suitability for LMICs is rated on a coarse scale of 1–5, with 1 indicating low suitability and 5 indicating high suitability. Scores were generated via qualitative assessment based on a range of factors (including cost, ease of use, need for sample shipping, need for expert analysis, invasiveness, etc).

IH, in-hospital; LFIA, lateral flow immunoassay; LMICs, low-income and middle-income countries; MS, mass spectrometry; OCT, optical coherence tomography; POC, point-of-care; TRL, technological readiness level.

Imaging capsules represent well validated technologies that have been widely used clinically. Both wireless capsule endoscopy and TC-OCT have the potential to provide information on villous morphology, and thus direct indications of absorptive surface area and damage, in a manner that is less invasive than standard endoscopy. Moreover, images can be processed in an automated way removing the requirement for analysis by an expert gastroenterologist.

A series of capsules for sampling or biopsy have been reported. The most useful for studies of EE will be those that provide location-specific sampling. Although several sampling capsules are under development (eg, see Ciuti *et al* 45), considerable validation is still required to demonstrate the reliability of sample collection and the location specificity. Once this work has been undertaken, however, opportunities in EE will be wide-ranging (although the requirement for sample analysis may necessitate either advanced facilities on-site or cold-chain shipping of samples to centralised analysis facilities). It is likely that all capsules will be best suited to deployment in clinics, however, it may be feasible to perform TC-OCT in POC settings as the device’s tether mitigates the risk of capsule retention. While further miniaturisation of capsules is highly likely to be necessary to facilitate widespread use in infants, it is noteworthy that even deployment in adults would provide important opportunities to improve understanding of the damaged gut in EE. Furthermore, given the progress made over recent years in incorporating additional functionality into endoscopic capsules (eg, see Sensing and combination (multifunctional) capsules section), further miniaturisation can be expected to be feasible if appropriate resources are made available.

The next technology listed in table 1 is optical spectroscopy, with both fluorescence and Raman spectroscopy highlighted. Transcutaneous fluorescence spectroscopy has been previously suggested as a POC tool for non-invasive assessment of intestinal permeability,^29^ where it has the potential to improve on current tests (eg, L:M tests) in terms of reliability, invasiveness, ease-of-deployment and even cost. A preliminary demonstration of this approach has been reported in rats,^60^ and human validation is currently under way.^61^ However, development of a handheld/wearable device that is suitable for POC deployment is still required.

Raman spectroscopy has the potential to provide advanced information across a number of domains of gut dysfunction (eg, assessment of the microbiota, quantification of bacterial translocation and analysis of breath samples). Handheld devices already exist, but improved sample preparation techniques will be required to allow effective POC deployment. In addition, further device development will be required for breath analysis, where analyte concentrations are typically low.

Portable sequencing technologies (eg, MinION/SmidgION, Oxford Nanopore Technologies) present an opportunity to quantitatively analyse the gut microbiome at the POC, potentially allowing EE measurements on a larger scale and in a longitudinal manner. Several hurdles remain, however, including achieving appropriate sample preparation, relatively high running costs, and the preparation of libraries for biomarker/metabolite quantitation. In addition, sequencing first requires sample collection. In the case of urine, blood or stool samples, this is feasible in POC settings, but collecting gut tissue biopsies is more challenging. Combining portable sequencing systems with the sampling/biopsy capsules discussed earlier may present a solution to this problem. While further development/validation of the sampling tools is necessary, the combination of these techniques could significantly improve understanding of the role of the microbiome and metabolome in undernutrition.

Breath tests represent another attractive technology in undernutrition because of their non-invasive sampling. Stable isotope breath tests are particularly attractive because of their potential to provide sensitive and specific assessment of functional parameters directly related to digestion and assimilation of essential nutrients, a domain of gut function that has been largely neglected in undernutrition. At present, samples are generally shipped to centralised facilities for analysis, despite availability of some POC systems (eg, based on IR spectroscopy^107^). Further development of these POC analysis systems is required as is identification of the most appropriate breath biomarker(s) for assessment of gut dysfunction in undernutrition. Nonetheless, breath tests present clear potential for biomarker analysis with non-invasive, longitudinal sample collection.

Knowledge gaps exist in our understanding of almost all aspects of immune function in undernutrition.^23 112^ Despite this, consistent relationships have been identified between inflammatory biomarkers and undernutrition prognoses and between pathogen carriage and EE. Assessing these (and other) markers at scale has the potential to improve understanding of the immunological components of undernutrition/EE and their association with enteropathogen carriage. Smartphone ELISA/LFIA are the only techniques for assessment of inflammatory biomarkers which are currently deployable at POC, but these are well validated and widely used in population studies of undernutrition. Adaptation of smartphones for microscopy, differential cell counting, flow cytometry and even lab-on-a-chip analysis is also under way, and such approaches hold promise for POC assessment of a broader range of immune functions.

Finally, we highlight miniaturised systems for metabolomic analysis. Portable mass spectrometers are under development but are unlikely to be deployable at scale in LMICs in the foreseeable future. Paper strip metabolomics, on the other hand, represents an approach that may be suitable for LMICs. Storing samples on paper strips circumvents the need for sample freezing and thus provides opportunities to collect metabolic profiles on large scales (although with a loss in sensitivity relative to fresh/frozen samples). Importantly, this technology already exists, and the only developmental work required will be in assessing the stability of biomarkers relevant to undernutrition.

Overall, the technologies listed in table 1 all have merit for the study of EE/undernutrition. While varying levels of development are required, the highlighted technologies provide information on elements of gut function ranging from villous structure and intestinal permeability to the composition of the microbiota and the expression of inflammatory biomarkers. As undernutrition results in multifaceted (and possibly context specific) aberrations, it is important that all (or many) of these domains are investigated simultaneously if we are to truly improve our understanding of this complex condition. The techniques highlighted here have the potential to do that across the wide-ranging domains, and technological development in one area may also facilitate new approaches in others. Thus, it is important that these techniques continue to be developed and validated for POC testing in parallel such that, in the future, multiple tools can be deployed for simultaneous assessment of complementary aspects of gut function. In turn, this will help to improve our understanding of EE/undernutrition and may also elucidate how to more effectively combat it.

**Key message: a multifaceted approach for a complex problem**

- A number of point-of-care diagnostic tools are available or under development for assessment of gut function in low-income and middle-income countries.
- These allow assessment of diverse aspects of gut function, including villous morphology, gut permeability, immune function, digestion, microbiota, etc.
- Crucially, these devices must be developed and deployed in combination if we are to truly improve understanding of the complex nature of gut dysfunction in environmental enteropathy (EE) and undernutrition.
- Finally, while this article focuses on EE/undernutrition, the highlighted technologies may also be useful in diagnosis and monitoring of a wide range of other gut disorders.

## Conclusions

Undernutrition is widespread in LMICs and accounts for approximately 45% of deaths in children under the age of 5.^1^ Importantly, provision of food does not reliably solve the problem, with both wasting and stunting being associated with a breakdown in gut function that limits essential nutrient availability and increases nutrient requirements. A variety of barriers to healthy gut function are evident in populations affected by EE and undernutrition, including chronic inflammation, alterations to the gut microbiota, and defects in digestion, gut barrier function and immune-mediated defence against pathogens. As such, addressing undernutrition requires a multifaceted approach, potentially including treatments to support gut health, sanitary/hygiene interventions, and even redesigning therapeutic feeds to be more efficiently digested and absorbed than current nutritional therapies. To achieve this, it will be necessary both to improve our understanding of undernutrition and to effectively monitor the impact of interventions over the course of hospital-based and community-based rehabilitation in a longitudinal manner. This will require tests and diagnostics that are minimal/non-invasive, that can assess multiple domains of gut function in parallel and that, in an ideal world, can be deployed in POC settings to allow validation in appropriately powered studies within affected communities.

In this article, we have reviewed a series of technologies that may be useful for this purpose. Each technology has been summarised in terms of the underlying principles and the potential for applications in undernutrition (ie, in terms of the information that could be offered). The devices range from optical imaging systems suitable for deployment in hospitals to POC sequencing tools for assessment of diverse biomarkers. Importantly, the information provided by the reviewed systems covers several domains of gut functional capacity, including villous morphology, intestinal permeability, bacterial translocation, inflammation, and even the state of the microbiota and metabolome. No one approach is likely to provide a ‘silver bullet’ to the challenge of understanding and monitoring gut dysfunction in undernutrition, largely due to the lack of existing information on the most informative indicators of gut pathogenesis. However, in combination, these technologies have the potential to improve our holistic understanding of gut function and to allow monitoring on large scales in affected communities. Thus, prioritising further development and deployment of the technologies reviewed here may help to accelerate the development of new/improved interventions to prevent undernutrition and promote nutritional recovery.
